# Supplementary material for: HIVprotI: an integrated web based platform for prediction and design of HIV proteins inhibitors
Source: J Cheminform. 2018 Mar 9;10:12. doi: 10.1186/s13321-018-0266-y (PMC5845081; doi:10.1186/s13321-018-0266-y)
Supplement: Supplementary file 2 — Additional file 2. Source code of HIVProtI web server. [file 13321_2018_266_MOESM2_ESM.zip › HIVprotI_Source-code/hivprotI/weka-3-6-6/doc/weka/gui/sql/ResultSetHelper.html]

ResultSetHelper


---


|  |  |  |
| --- | --- | --- |
| **Overview** \| **Package** \| **Class** \| **Tree** \| **Deprecated** \| **Index** \| **Help** \| **Weka's home** | |  |
| **PREV CLASS**   **NEXT CLASS** | **FRAMES**    **NO FRAMES**     **All Classes** |  |
| SUMMARY: NESTED \| FIELD \| CONSTR \| METHOD | DETAIL: FIELD \| CONSTR \| METHOD |  |


---


## weka.gui.sql Class ResultSetHelper

```
java.lang.Object
  weka.gui.sql.ResultSetHelper
```

---

``` public class ResultSetHelper extends java.lang.Object ```

Represents an extended JTable, containing a table model based on a ResultSet
and the corresponding query.

**Version:**
:   $Revision: 7043 $

**Author:**
:   FracPete (fracpete at waikato dot ac dot nz)

---

**Constructor Summary**

`ResultSetHelper(java.sql.ResultSet rs)`
  
          initializes the helper, with unlimited number of rows.

`ResultSetHelper(java.sql.ResultSet rs,
int max)`
  
          initializes the helper, with the given maximum number of rows (less than
1 means unlimited).


| **Method Summary** | |
| --- | --- |
| `java.lang.Object[][]` | `getCells()`             returns an 2-dimensional array with the content of the resultset, the first dimension is the row, the second the column (i.e., getCells()[y][x]). |
| `java.lang.Class[]` | `getColumnClasses()`             returns the classes for the columns. |
| `int` | `getColumnCount()`             returns the number of columns in the resultset. |
| `java.lang.String[]` | `getColumnNames()`             returns an array with the names of the columns in the resultset. |
| `int` | `getMaxRows()`             the maximum number of rows to retrieve, less than 1 means unlimited. |
| `boolean[]` | `getNumericColumns()`             returns an array that indicates whether a column is numeric or nor. |
| `java.sql.ResultSet` | `getResultSet()`             the underlying resultset. |
| `int` | `getRowCount()`             returns the number of rows in the resultset. |
| `boolean` | `hasMaxRows()`             whether a limit on the rows to retrieve was set. |
| `static boolean` | `typeIsNumeric(int type)`             returns whether the SQL type is numeric (and therefore the justification should be right). |
| `static java.lang.Class` | `typeToClass(int type)`             Returns the class associated with a SQL type. |

**Methods inherited from class java.lang.Object**

`equals, getClass, hashCode, notify, notifyAll, toString, wait, wait, wait`

**Constructor Detail**

### ResultSetHelper

```
public ResultSetHelper(java.sql.ResultSet rs)
```

:   initializes the helper, with unlimited number of rows.

    **Parameters:**: `rs` - the resultset to work on

---


### ResultSetHelper

```
public ResultSetHelper(java.sql.ResultSet rs,
                       int max)
```

:   initializes the helper, with the given maximum number of rows (less than
    1 means unlimited).

    **Parameters:**: `rs` - the resultset to work on: `max` - the maximum number of rows to retrieve


**Method Detail**

### getResultSet

```
public java.sql.ResultSet getResultSet()
```

:   the underlying resultset.

    :   **Returns:**: the resultset

---


### getColumnCount

```
public int getColumnCount()
```

:   returns the number of columns in the resultset.

    :   **Returns:**: the number of columns

---


### getRowCount

```
public int getRowCount()
```

:   returns the number of rows in the resultset. If -1 then the number of
    rows couldn't be determined, i.e., the cursors aren't scrollable.

    :   **Returns:**: the number of rows, -1 if it wasn't possible to determine

---


### getColumnNames

```
public java.lang.String[] getColumnNames()
```

:   returns an array with the names of the columns in the resultset.

    :   **Returns:**: the column names

---


### getNumericColumns

```
public boolean[] getNumericColumns()
```

:   returns an array that indicates whether a column is numeric or nor.

    :   **Returns:**: the numeric columns

---


### getColumnClasses

```
public java.lang.Class[] getColumnClasses()
```

:   returns the classes for the columns.

    :   **Returns:**: the column classes

---


### hasMaxRows

```
public boolean hasMaxRows()
```

:   whether a limit on the rows to retrieve was set.

    :   **Returns:**: true if there's a limit

---


### getMaxRows

```
public int getMaxRows()
```

:   the maximum number of rows to retrieve, less than 1 means unlimited.

    :   **Returns:**: the maximum number of rows

---


### getCells

```
public java.lang.Object[][] getCells()
```

:   returns an 2-dimensional array with the content of the resultset, the first
    dimension is the row, the second the column (i.e., getCells()[y][x]).
    Note: the data is not cached! It is always retrieved anew.

    :   **Returns:**: the data

---


### typeToClass

```
public static java.lang.Class typeToClass(int type)
```

:   Returns the class associated with a SQL type.

    :   **Parameters:**: `type` - the SQL type **Returns:**: the Java class corresponding with the type

---


### typeIsNumeric

```
public static boolean typeIsNumeric(int type)
```

:   returns whether the SQL type is numeric (and therefore the justification
    should be right).

    :   **Parameters:**: `type` - the SQL type **Returns:**: whether the given type is numeric


---


|  |  |  |
| --- | --- | --- |
| **Overview** \| **Package** \| **Class** \| **Tree** \| **Deprecated** \| **Index** \| **Help** \| **Weka's home** | |  |
| **PREV CLASS**   **NEXT CLASS** | **FRAMES**    **NO FRAMES**     **All Classes** |  |
| SUMMARY: NESTED \| FIELD \| CONSTR \| METHOD | DETAIL: FIELD \| CONSTR \| METHOD |  |


---
